# Platelet to lymphocyte ratio was a risk factor in Perthes disease

**DOI:** 10.1038/s41598-023-32000-0

**Published:** 2023-03-28

**Authors:** TianTian Wang, XiaoLin Luo, BoXiang Li, Qian Huang, JianHong Liu, ShengPing Tang, Yun Liu, RongBin Lu, ShiJie Liao, XiaoFei Ding

**Affiliations:** 1 Department of Orthopedics, Ordos Central Hospital, 23 Ekin Hollow West Street, Ordos, 017000 China; 2 The First Affiliated Hospital of Guangxi Medical University, Nanning, 530000 China

**Keywords:** Biomarkers, Diseases

## Abstract

The study was aimed to determine the relationship between PLR (platelet to lymphocyte ratio) and the lateral pillar classification of Perthes disease, and to provide an alternative index for clinical diagnosis. In addition, the association of the PLR with the necrosis stage of Perthes disease was also explored. This was a retrospective study. 74 children with Perthes disease and 60 children in the healthy control group without femoral head necrosis in our hospital from 2012 to 2021 were collected. The general data and clinical parameters were collected from the hospital information system. The modified herring lateral pillar classification was collected for the fragmentation stage case group and the PLR, NLR (neutrophil to lymphocyte ratio), LMR (lymphocyte to monocyte ratio) and PNR (platelet to neutrophil ratio) were calculated. The cases were divided into four groups, herring A and B were group I, herring B/C and C were group II, the healthy control group was group III, and the necrosis stage was group IV. The hematological indexes (NLR, PLR, LMR, PNR) of children at different stages were statistically analyzed. Group I consisted of 36 patients, with an average age of 7.4 ± 2.0 years (3–11 years). Group II consisted of 23 patients, with an average age of 7.4 ± 1.9 years (4–12 years). Group III consisted of 60 patients, with a mean age of 7.4 ± 2.7 years (4–13 years). Group IV consisted of 15 patients, with an average age of 6.4 ± 1.7 years (3–10 years). The average values of PLR in groups I, II, III and IV were 131.98 ± 47.44, 122.19 ± 37.88, 102.46 ± 30.68 and 128.90 ± 28.11, respectively. It's worth noting that there was statistically significant difference among groups I, II and III (*P* = 0.003). The optimal threshold of PLR was 130.25, the sensitivity was 45.8% and the specificity was 85%. PLR was also significantly different between groups III and group IV. PLR was higher in Herring A and B classifications than in Herring B/C and C classifications. PLR had certain diagnostic value in both the necrosis stage and fragmentation stage as a risk factor.

## Introduction

Legg–Calvé–Perthes disease (LCPD) is a kind of child femoral head epiphysis ischemic necrosis syndrome and a common idiopathic hip disease in children. It is more common in boys than girls aged 4 to 8 years, with an incidence of about 29 per 100,000^1^. The pathological process consists of necrosis stage, fragmentation stage, reossification stage, and healed stage. The involvement of the femoral head in the later stages of the disease can leave varying degrees of deformity, affecting the physical and mental health of the child. The pathogenesis of LCPD is still unknown, and the insidious onset and lack of specific diagnostic markers make early diagnosis and intervention difficult^2^. Therefore, it is particularly important to find convenient and effective early diagnostic markers.

A growing number of studies now show that inflammation is closely associated with LCPD. Perthes disease is often associated with hip synovitis on the affected side. Kim et al. ^3^ showed that Perthes disease is related to the increase in the level of pro-inflammatory cytokine in synovial fluid. Scholars also found that necrotic bone stimulates macrophage inflammation through TLR4 in the piglet model of Perthes disease^4^. Our previous studies also showed that plasma in Perthes patients can induce endothelial cell apoptosis and further cause endothelial dysfunction ^5^. However, sample collection of hip synovial fluid in clinics is difficult. Finding some easily accessible indicators of inflammation can help us diagnose and treat early in order to improve the prognosis of the disease.

In recent years, some hematological parameters such as NLR, PLR, NPR and LMR have been considered as representative markers of systemic inflammatory response and are widely used in several cardiovascular diseases, chronic inflammatory diseases and malignancies^6–10^, but are less studied in Perthes disease. Meanwhile, the Herring lateral pillar classification, a method of imaging assessment during fragmentation stage, is widely used in clinical practice because of its predictive role in long-term prognosis^11^. Therefore, the objective of this research was to determine the relationship between inflammatory indicators and the lateral pillar classification during fragmentation stage of LCPD. In addition, the relationship between these indicators and the necrosis stage of LCPD was also explored.

## Materials and methods

### General information

This study retrospectively analysed 74 patients (59 in fragmentation stage and 15 in necrosis stage) with Perthes disease who were hospitalised in the Department of Orthopaedic Trauma Hand Surgery of the First Affiliated Hospital of Guangxi Medical University from January 1, 2012 to July 31, 2021. The inclusion criteria included the following: 1. children with Perthes disease and radiographic images at fragmentation stage and necrosis stage; 2. complete data on preoperative blood routine examination; and 3. complete data on pelvic plain film. The exclusion criteria included the follows: 1. patients with acute upper respiratory tract infection that may change the levels of inflammatory indicators; 2. patients with congenital femoral head malformation and other causes of femoral head necrosis, such as infection, trauma, hormone DDH treatment, other hip lesions, diabetes, cardiovascular disease, kidney disease, coagulation abnormalities and malignant tumours; and 3. patients with bilateral femoral head necrosis.

### Research methods

The collected data included the general information (age, gender, height and weight) and the modified herring lateral pillar classification of the lateral column of the patients. Haematologic markers NLR, PLR, LMR, PNR albumin and alkaline phosphatase were recorded. Data, except lateral column grading, were also collected from 60 healthy controls matched by age (< 1 year difference) and sex during the same period. The other exclusion criteria were femur head necrosis, epiphyseal dysplasia, femoral fracture and other reasons. Patients with LCPD at the fragmentation stage were split into two groups on the basis of expected prognosis: group I consisted of patients of Herring A and B with good outcome, group II consisted of Herring B/C and C patients with poor outcome and group III consisted of healthy children. Patients in the necrosis stage was group IV. Haematologic indicators were analysed for all patients.

### Methods of statistics

SPSS 24.0 (IBM, USA) software was used for statistics. Age and BMI were analyzed by ANOVA. Chi square test and Fisher exact probability method were used for sex and sex. As the data volume is more than 50, the normality of continuous data is tested by Kolmogorov Smirnov test (K-S test), and the comparison of multiple continuous independent samples is tested by Kruskal Wallis test. When p < 0.05, the difference was considered statistically significant. Least Significant Difference method was used for pairwise comparison of groups I, II, and III.

### Ethical statement

All methods were carried out in accordance with relevant guidelines and regulations. The Ethics Committee of the First Affiliated Hospital of Guangxi Medical University approved all our experimental protocols. Informed consent was obtained from the parents of all participants, and published in an online open access publication.

## Results

**Figure 1:**
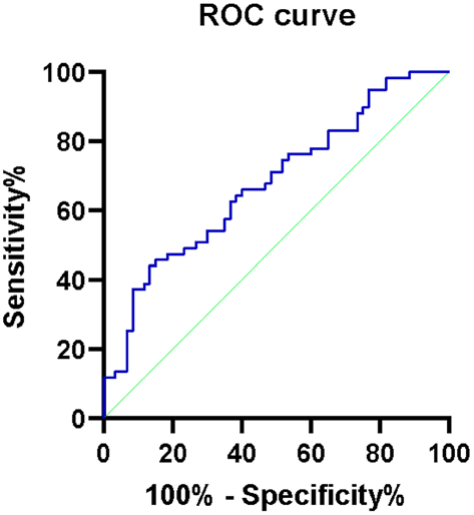
Prediction of receiver operating characteristic (ROC) curves in Perthes disease with PLR. The area under ROC curve (AUC) was 0.679, *P* = 0.001.

The baseline data of each group are presented in Table 1. The comparison of inflammatory indicators between LCPD in fragmentation stage and the control group is shown in Table 2. The PLR in patients with fragmentation stage was higher than the control group, with statistically significant difference (*P* = 0.001). The NLR in case group was higher than the control group, with no statistically significant difference (*P* = 0.085), nevertheless. The NLR, PLR, LMR and PNR values in groups I, II and III are shown in Table 3. The PLR value in group I was exceeding the two other groups (*P* = 0.003). The *P* values were successively 0.334, 0.036 and 0.0000 between groups I and II, II and III, and I and III, indicating that grouping I and II based on the classification of lateral pillar classification had no statistical significance for the prognosis judgment, and could not be used as a predictor to judge the prognosis of Perthes disease. Comparison of inflammatory indicators between LCPD in necrosis stage and group III was shown in Table 4. Results showed that PLR was not only significantly different between the fragmentation stage and the control group, but also significantly higher in patients in the necrosis stage. We employ ROC curve analysis to evaluate the most favorable threshold value and Youden index for PLR to predict Perthes disease in Fig. 1. When the PLR Youden index was about 0.3076 and the threshold was 130.25, the sensitivity of Perthes disease was 45.8%, the specificity was 85% and the area on the underside of the curve was 0.679.

**Table 1:** Basic information.

Groups	Group I	Group II	Group III	Total	*P*-value
Patients	36	23	60	119	–
Age (years)	7.4 ± 2.0(3–11)	7.4 ± 1.9(4–12)	7.4 ± 2.7(4–13)	7.4 ± 2.4(3–13)	0.998
Sex Male	33(91.7%)	22(95.7%)	50(83.3%)	105(88.2%)	0.306
Laterality Left	10(27.8%)	10(43.5%)	–	20(33.9%)	0.214
BMI	16.2 ± 3.2	17.7 ± 3.1	16.0 ± 2.7	16.4 ± 3.0	0.072

**Table 2:** Comparison of inflammatory indexes between Perthes group and control group.

	NLR	PLR	LMR	1/PNS
Perthes	1.41 ± 0.64	128.16 ± 43.88	5.65 ± 1.61	100.50 ± 36.92
Control	1.22 ± 0.54	102.46 ± 30.68	6.16 ± 2.10	93.60 ± 30.58
*P*-value	0.085	0.001*	0.294	0.470

Significant values are in bold.

*Statistically significant.

**Table 3:** Comparison of inflammatory markers in each group.

	NLR	PLR	LMR	1/PNS
Group I	1.49 ± 0.73	131.98 ± 47.44	5.64 ± 1.73	99.55 ± 37.55
Group II	1.30 ± 0.46	122.19 ± 37.88	5.66 ± 1.43	102.00 ± 36.68
Group III	1.22 ± 0.54	102.46 ± 30.68	6.16 ± 2.10	93.60 ± 30.58
*P*-value	0.198	0.003*	0.569	0.723

Significant values are in bold.

*Statistically significant.

**Table 4:** Comparison of inflammatory indexes between LCPD in necrosis stage and control group.

	NLR	PLR	LMR	PNR
Perthes	1.58 ± 0.78	128.90 ± 28.11	5.27 ± 3.15	98.45 ± 42.44
Control	1.22 ± 0.54	102.46 ± 30.68	6.16 ± 2.10	93.60 ± 30.58
*P* value	0.037*	0.003*	0.187	0.614

Significant values are in bold.

*Statistically significant.

## Discussion

PLR is a simple and economical marker of systemic inflammatory response, commonly used to reflect acute inflammation and pre-thrombotic state changes, and is elevated in rheumatic diseases and chronic inflammatory diseases such as systemic lupus erythematosus and rheumatoid arthritis^12,13^. The objectives of this study were to determine the relationship between the lateral pillar classification and PLR during the fragmentation stage of LCPD and to further analyse the relationship between these inflammatory indicators and the necrotic stage. The results showed that PLR was higher in the lateral pillar classification A and B groups with higher inflammatory response than in the B/C and C groups. PLR was significantly higher than the control group in the necrosis stage and fragmentation stage of LCPD, which were easily missed and misdiagnosed, and the difference was statistically significant.

The clinical manifestations of LCPD were identified more than 100 years ago by Legg in America, Calvé from France along with Perthes from Germany^14–16^. However, imaging manifestations, such as necrosis and early fragmentation, are not obvious at the early stages of Perthes; as such, the disease is easy to be missed and misdiagnosed. Once the precious stage of early treatment is missed, femoral head deformities, such as hip planus and osteoarthritis, will occur. Therefore, early diagnosis and prognostic indicators can guide the clinical diagnosis and treatment of Perthes disease. Current prognostic indicators in LCPD include age at clinical onset, degree of epiphyseal involvement, degree of femoral head deformity, disease stage and the modified herring lateral pillar classification^17^. The modified herring lateral pillar classification is a commonly used and recognised prognostic indicator in clinical practice. Therefore, exploring the association of hematological indicators and the lateral pillar classification is expected to find early diagnostic and prognostic indicators that can guide the clinical management of Perthes' disease.

Previous studies showed that NLR is elevated in children with femoral head necrosis, and NLR in Herring A and B groups with better prognosis was lower than that in Herring B/C and C groups with poor prognosis^18^. Hence, NLR can predict the prognosis of patients with LCPD to some extent. In this study, the PLR value of group I representing herring type A and B with good prognosis was higher than that of group II representing herring type B / C and C with poor prognosis. Despite the lack of statistical differences, it can still be assumed that PLR values are higher in the herring type A and B with higher inflammatory response than in herring type B / C and C, which to a certain extent indicates that the inflammatory index in the early stage of fragmentation is higher than that in the middle and late stage of fragmentation. In the late stage of fragmentation, the femoral head is often already partially repaired. Kim et al.^19^ found that inflammatory cytokine in synovial fluid during the active phase of the disease, increased significantly in the early stage of fragmentation through MRI evaluation of synovitis and fluid volume of 28 patients and measurement of cytokines in 13 synovial fluid. The results of this study are consistent with those of Kim et al.

This study also indicates that PLR was associated with the diagnosis of Perthes disease, while NLR, LMR and PNR were not statistically significant in the diagnosis and prediction of the disease. Femoral head deformity starts in the necrosis stage and progresses in the fragmentation stage, in which excessive activation of osteoclasts is an important pathological change^20^. Non-classical activation pathways via inflammatory factor-mediated osteoclasts may play a role in this process. Inhibition of this osteoclast activation pathway using exogenous recombinant osteoprotegerin in animal models significantly alleviates femoral head deformities^21^. These results all indicate that inflammation may play an important role in the early stages of the disease and were confirmed in our study. ROC curve was applied to compare the diagnosis value of PLR in Perthes disease. The maximum area under the curve of PLR was 0.679, the sensitivity was 45.8% and the specificity was 85%. This result shows that PLR, an inflammatory index, has certain diagnostic value in Perthes disease.

However, in this retrospective study, the sample size was small, and the sensitivity and specificity of PLR were not high enough. Therefore, future prospective multicenter studies with large sample sizes are needed to analyze blood samples at different stages.

## Conclusion

PLR was higher in Herring A and B classifications than in Herring B/C and C classifications, and had some diagnostic value in both the necrosis stage and fragmentation stage, which are difficult to diagnose clinically. High PLR was a risk factor for the disease.

## Data Availability

All data generated or analyzed during this study are included in this published article.
